# The CHIP-Family study to improve the psychosocial wellbeing of young children with congenital heart disease and their families: design of a randomized controlled trial

**DOI:** 10.1186/s12887-018-1183-y

**Published:** 2018-07-12

**Authors:** Malindi van der Mheen, Ingrid M. van Beynum, Karolijn Dulfer, Jan van der Ende, Eugène van Galen, Jorieke Duvekot, Lisette E. Rots, Tabitha P. L. van den Adel, Ad J. J. C. Bogers, Christopher G. McCusker, Frank A. Casey, Willem A. Helbing, Elisabeth M. W. J. Utens

**Affiliations:** 1 Department of Child and Adolescent Psychiatry/Psychology, Erasmus MC – Sophia Children’s Hospital, KP-2865, Wytemaweg 8, 3015 CN Rotterdam, The Netherlands; 2 Department of Pediatric Cardiology, Erasmus MC – Sophia Children’s Hospital, Rotterdam, The Netherlands; 3 Dutch Patient Association for Congenital Heart Disease, Maarssen, The Netherlands; 4 Psychosocial Care Unit, Erasmus MC – Sophia Children’s Hospital, Rotterdam, The Netherlands; 5 Department of Pediatric Physiotherapy, Erasmus MC – Sophia Children’s Hospital, Rotterdam, The Netherlands; 6 Department of Thoracic Surgery, Erasmus MC, Rotterdam, The Netherlands; 7 School of Applied Psychology, University College Cork, Cork, Ireland; 8 Department of Pediatric Cardiology, Royal Belfast Hospital for Sick Children, Belfast, Ireland; 9 Research Institute of Child Development and Education, University of Amsterdam, Amsterdam, The Netherlands; 10 Academic Centre for Child and Adolescent Psychiatry the Bascule/Department of Child and Adolescent Psychiatry, Academic Medical Centre, Amsterdam, The Netherlands

**Keywords:** Congenital heart defects, Children, Families, Psychosocial wellbeing, Intervention, CHIP

## Abstract

**Background:**

Children with congenital heart disease (CHD) are at increased risk for behavioral, emotional, and cognitive problems. They often have reduced exercise capacity and participate less in sports, which is associated with a lower quality of life. Starting school may present more challenges for children with CHD and their families than for families with healthy children. Moreover, parents of children with CHD are at risk for psychosocial problems. Therefore, a family-centered psychosocial intervention for children with CHD when starting school is needed. Until now, the ‘Congenital Heart Disease Intervention Program (CHIP) – School’ is the only evidence-based intervention in this field. However, CHIP-School targeted parents only and resulted in non-significant, though positive, effects as to child psychosocial wellbeing. Hence, we expanded CHIP by adding a specific child module and including siblings, creating the CHIP-Family intervention. The CHIP-Family study aims to (1) test the effects of CHIP-Family on parental mental health and psychosocial wellbeing of CHD-children and to (2) identify baseline psychosocial and medical predictors for the effectiveness of CHIP-Family.

**Methods:**

We will conduct a single-blinded randomized controlled trial comparing the effects of CHIP-Family with care as usual (no psychosocial intervention). Children with CHD (4–7 years old) who are starting or attending kindergarten or primary school (first or second year) at the time of first assessment and their families are eligible. CHIP-Family consists of a separate one-day workshop for parents and children. The child workshop consists of psychological exercises based on the evidence-based cognitive behavioral therapy Fun FRIENDS protocol and sports exercises. The parent workshop focuses on problem prevention therapy, psychoeducation, general parenting skills, skills specific to parenting a child with CHD, and medical issues. Approximately 4 weeks after the workshop, parents receive an individual follow-up session. The baseline (T1) and follow-up assessment (T2 = 6 months after T1) consist of online questionnaires filled out by the child, parents, and teacher (T2 only). Primary outcome measures are the CBCL for children and the SCL-90-R for parents.

**Discussion:**

This trial aims to test the effects of an early family-centered psychosocial intervention to meet the compelling need of young children with CHD and their families to prevent (further) problems. If CHIP-Family proves to be effective, it should be structurally implemented in standard care.

**Trial registration:**

Dutch Trial Registry; NTR6063 on 23 August, 2016.

## Background

Children with congenital heart disease (CHD) are at elevated risk for behavioral, emotional, and cognitive problems in childhood [1, 2], adolescence, and adulthood [3]. Previous cohort studies from our research group have indicated that CHD-children are two times more likely to develop psychopathology than healthy children (16–27% versus 10% in the general population) - irrespective of the type of cardiac defect [4, 5]. Especially internalizing behavior problems, problems with social contacts, and reduced quality of life have been reported [6]. Moreover, neuropsychological problems and intellectual impairments are well known in these children [7, 8] and elevated percentages of CHD-children attending special education (24% versus 4% in norm) have been reported [3]. The most common morbidity affecting the quality of life in school-aged children with CHD is the combination of behavioral/emotional problems, developmental delay, and school difficulties [9]. Such problems can have long-term consequences: two long-term studies have shown that adults with CHD overall had a lower occupational and educational status compared with the general population [10, 11]. Furthermore, children with CHD often have reduced exercise capacity and participate less in exercise and sports, which has been associated with a lower quality of life [12]. It has been shown that participation in an exercise program improves quality of life of children with CHD [13].

In addition, parental factors play a crucial role in children’s psychosocial wellbeing [2, 14–16]. Maternal mental health and worry have appeared to be more important predictors of psychosocial wellbeing of children with CHD than illness severity [2, 17, 18]. Unfortunately, parents of children with CHD are also at risk for psychosocial problems themselves (e.g. anxiety, depression; 1 year prevalence 7–22%) [19].

Considering the above and the fact that milestones such as starting kindergarten and primary school present more challenges for children with CHD and their parents than for families with healthy children [20], a family-based psychosocial intervention tailored to their needs when starting school is required [19, 21, 22]. This need has also been expressed by parents and patients [21, 23]. Through such an intervention, psychosocial problems of children with CHD and their parents may be recognized, reduced or prevented. In addition, school functioning, emotional resilience, and sports participation of these children can be improved [21, 24]. Until now, the only evidence-based intervention in this field is the Congenital Heart Disease Intervention Program (CHIP) – School [2]. The CHIP-School study aimed to promote psychosocial wellbeing of preschoolers with CHD indirectly by providing an intervention for their parents. CHIP-School resulted in significant gains in maternal mental health, reduced perceived strain on the family, and less school absence of the child. As to child psychosocial wellbeing, only a non-significant, though positive, trend was found [2].

A limitation of CHIP-School was that a separate child module was not included. Therefore, in collaboration with the original authors of the previous CHIP intervention, we have translated, extended and modified CHIP, by adding a tailored child module for CHD-children and their siblings. The child module includes evidence-based cognitive behavioral exercises [25] and sports exercises. The newly developed CHIP-Family is a psychosocial intervention for 4- to 7-year-old children who have undergone at least one medical intervention for CHD and are starting or attending kindergarten or primary school (first or second year) and their families.

The aim of this study is (1) to test the effects of CHIP-Family on parental mental health and psychosocial wellbeing of CHD-children who are starting or attending kindergarten or primary school and to (2) identify baseline psychosocial and medical predictors for the effectiveness of CHIP-Family.

## Methods

This study is a single-center, single-blinded randomized controlled trial (RCT) comparing the effects of the CHIP-Family intervention with care as usual (CAU; regular medical treatment) on mental health of parents and psychosocial wellbeing of young children with CHD. This RCT is designed according to the CONSORT guidelines [26].

### Inclusion and exclusion criteria

Over a one-year period (September 2016 – September 2017) children and their families living in the Netherlands will be recruited. Eligible are all children who (1) underwent at least one invasive procedure (catheter intervention or surgery) for CHD and (2) are starting or attending kindergarten or primary school (first or second year) at the time of first assessment (as the children are approximately 4–7 years old). Exclusion criteria are: (1) child’s intellectual impairment (IQ < 70) as ascertained by previous standardized assessment or diagnosed by a clinician, (2) insufficient mastery of the Dutch language, and (3) prematurely born children (gestational age at birth < 37 weeks) with no other CHD than a patent ductus arteriosus.

### Recruitment and procedure

Parents of 4- to 7-year-old children who receive treatment at the department of pediatric cardiology of the Erasmus Medical Center – Sophia Children’s Hospital and eligible members of the Dutch Patient Association for Congenital Heart Disease whose children receive treatment in a cardiac centre in the Netherlands will receive an information leaflet explaining the purpose and procedures of the study. Before inclusion, parents will receive a verbal explanation of the trial. After obtaining written parental informed consent, patients are randomly allocated to the CHIP-Family intervention or CAU group. To avoid a delay of more than 1 month between baseline assessment and the intervention, patients are randomized prior to the baseline assessment. Patients are allocated to the CHIP-Family intervention or CAU group by means of block randomization, performed by an independent researcher. Randomization will be stratified by CHD severity (limited to no residual heart defects or moderate to severe residual heart defects [after medical intervention]; see Table 1) and school year (kindergarten or primary school). To avoid bias, the researcher performing the assessments and analyses will be blinded. Considering the nature of the CHIP-Family intervention, it is not possible to blind the participants and the health care professionals providing the intervention.

**Table 1:** Stratification factor “CHD severity”

Type 1	Type 2
Limited to no residual heart defects after medical intervention	Moderate to severe residual heart defects after medical intervention
Atrial Septal Defect (ASD)	ALCAPA (Anomalous Left Coronary Artery from the Pulmonary Artery)
Patent Ductus Arteriosus	Aortic Valve Stenosis
Pulmonary valve stenosis	Atrioventricular Septal Defect (AVSD)
Total Anomalous Pulmonary Venous Connection	Coarctation of the Aorta
Ventricular Septal Defect (VSD)	Complex Biventricular (e.g. Truncus Arteriosus, aortic arch defects)
	Double Inlet Ventricle – Fontan circulation
	Ebstein’s Anomaly
	Subvalvular Aortic Stenosis
	Tetralogy of Fallot (TOF)
	TOF with MAPCA (Main Aorta to Pulmonary Connecting Artery)
	Transposition of the Great Arteries

### Intervention

CHIP-Family consists of a parent module and a child module. Parents and children participate in a separate, but simultaneously given, 6-h group workshop. An overview of the content of the workshops is given in Table 2. Over the course of a 11-month period (Nov. 2016 – Sept. 2017) 11 workshops will be given to 3 to 5 families per workshop.

**Table 2:** Outline of the CHIP-Family workshops

Health care professional(s)	Content
Parent workshop	
Psychologists	• Problem prevention therapy [48]. A DO ACT acronym is applied: **D**efine problem and turn into a specific goal; **O**ption brainstorm; **A**ssess pros and cons of various options; **C**hoose a strategy; **T**ake action and evaluate
	• Psychoeducation
	• General parenting skills
	• Specific parenting skills for children with CHD
Pediatric cardiologist	• Information on medical diagnoses, treatments, future issues (e.g., career, pregnancy), insurance, and healthy living (e.g., sports, diet)
Child workshop	
Psychologists	• Relaxation
	• Promoting autonomy
	• Strengthening self-esteem
	• Making friends
	• Problem solving skills
	• Positive thinking
Physiotherapists	• Playful, age-attuned sports exercises: warming-up, fitness, gross motor skills, balance, aiming, and catching

### Parent module

The parent module is based on the evidence-based CHIP-School protocol [2].

#### Workshop

The parent workshop focuses on problem prevention therapy, psychoeducation, general parenting skills, skills specific to parenting a child with CHD (given by two senior psychologists with expertise in the field; 4 h), and medical issues (given by a pediatric cardiologist; 1 h). The lunch break (1 h) offers families more opportunity to interact and share (similar) experiences. During the workshop, parents receive a manual which contains an overview of the topics that will be covered during the workshop and a home assignment on problem prevention therapy. Parents also receive handouts and a teacher information leaflet.

#### Follow-up booster session

Approximately 4 weeks after the workshop, parents receive an individual follow-up booster session with a psychologist who was present during the parent workshop and a psychologist who was present during the child workshop. Questions or worries that may have come up after the workshop regarding their child with CHD or their family members are discussed. Also, aspects of the workshop which have been (most) helpful for parents and will be helpful in the future are reviewed. Moreover, the session focuses on the problem prevention home assignment and on how to promote future use of problem prevention therapy.

### Child module

To normalize participation in the workshop and to stimulate practice at home, each child is allowed to bring a 4- to 10-year-old sibling or friend. The psychological exercises (given by two junior psychologists; 4 h) are based on the evidence-based cognitive behavioral therapy Fun FRIENDS protocol [25]. The exercises are provided in a playful manner and focus on regulating emotions, relaxation, promoting autonomy, strengthening self-esteem, making friends, problem solving skills, and positive thinking. The playful, age-attuned sports exercises (given by a physiotherapist and assistant physiotherapist; 1 h) are based on a standardized training program. Previous research has shown that these exercises are effective in improving the quality of life in children with CHD [13].

### Training and protocol adherence

CHIP-Family is performed in a standardized manner. Prior to the workshops, four senior and five junior psychologists receive a one-day CHIP-training by developmental psychologists Prof. Dr. McCusker and Dr. Doherty, developers of the original CHIP-protocol. To ensure consistency, the same senior and junior psychologist will be present at each workshop. In both the parent and child workshops, another psychologist will be present. Master’s students in Psychology will assess treatment integrity during the parent and child workshop through a standardized form. Follow-up sessions are audiotaped and treatment integrity is assessed through a standardized form afterwards.

### Outcome measures

An overview of all variables and questionnaires per assessment moment is given in Table 3. All questionnaires are (inter)nationally validated and Dutch normative data is available. Children and their families are enrolled into the study in groups of 6 to 10 families (3 to 5 families in the CHIP-group and 3 to 5 families in the CAU group). In both the CHIP-Family and the CAU condition, the first assessment will take place within 2 weeks before the CHIP-Family intervention (T1) and the follow-up post-assessment (T2) will take place 6 months after T1. Patients who are randomized into the CHIP-Family intervention group complete a social validity questionnaire assessing satisfaction with regards to the CHIP-program within 2 weeks after the intervention and at T2. All questionnaires are completed at home through a secure website.

**Table 3:** Assessment instruments and moments of assessment

Instrument	Variable	Assessment moment		
		T1	Direct follow-up	T2
Primary outcomes				
Child Behavior Checklist (CBCL) [32]	Child behavioral/emotional problems	M, F		M, F
Symptom Checklist-90-Revised (SCL-90-R) [29]	Parental mental health	M, F		M, F
Secondary outcomes				
Rotterdam Quality of Life interview [30]	School days sick/absent	M, F		M, F, T
Rotterdam Knowledge Questionnaire [31]	Disease-specific knowledge and illness perception	M, F		M, F
Teacher Report Form (TRF) [32]	School functioning	-		T
Behavior Rating Inventory of Executive Functioning (BRIEF) [34, 35] or BRIEF-Preschool Version (BRIEF-P) [36]	Executive functioning	M, F		M, F, T
Adjusted Groningen Enjoyment Questionnaire [37]	Sports participation, enjoyment of physical activity	M, F		M, F, T
2 sports-related questions	Sports participation, enjoyment of physical activity	C		C
Penn State Worry Questionnaire (PSWQ) [49]	Parental worry	M, F		M, F
Nijmeegse Ouderlijke Stress Index verkort (NOSIK) [40]	Parental stress	M, F		M, F
Stress thermometer (DT-P) [50]	Parental stress	M, F		M, F
Child Health Questionnaire (CHQ-PF50) [51]	Quality of life of child and sibling	M, F		M, F
Short-form (36) Health Survey (SF-36) [52]	Quality of life of parents	M, F		M, F
Family Assessment Device, general functioning subscale (FAD) [53]	Family functioning	M, F		M, F
Medical record	Medical consumption	R		R
Social validity questionnaire	Satisfaction, attendance, and completion of CHIP-Family	–	M, F	M, F
Predictor variables				
Rotterdam Quality of Life interview [30]	Demographic variables	M, F		–
Medical record	Cardiac diagnosis	R		R
Life event subscale of the Cognitive Emotion Regulation Questionnaire, child version (CERQ-k) [44]	Life events	M, F		M, F

*M* Mother, *F* Father, *C* Child, *T* Teacher, *R* Medical records

T1 = baseline, Direct follow-up = within 2 weeks after CHIP-Family intervention (only for participants in intervention group), T2 = follow-up, 6 months after T1

#### Primary outcomes

##### Child behavioral/emotional problems

The problem section of the Child Behavior Checklist (CBCL) [27] 1,½-5 (100 items; for 4- and 5-year-olds) and CBCL/6–18 (120 items; for 6- and 7-year-olds) will be used to obtain standardized parent reports of emotional and behavioral problems in their child. Response categories range from 0 to 2, with higher scores indicating more emotional and/or behavioral problems. Adequate reliability and validity have been reported [28].

##### Parental mental health

The Symptom Checklist-90-Revised (SCL-90-R) [29] is a self-report scale (90 items; response categories: 1–5, higher score indicates more symptoms) which assesses 9 primary symptom dimensions: Somatization, Obsessive-Compulsive, Interpersonal Sensitivity, Depression, Anxiety, Hostility, Phobic Anxiety, Paranoid Ideation, and Psychoticism. Adequate reliability and validity have been reported for the Dutch version [29].

#### Secondary outcomes

##### School days sick/absent

Through the Rotterdam Quality of Life interview [30] parents and teachers will be asked how many days the child was absent from school and what the reasons for absence were.

##### Disease-specific knowledge and illness perception

The Rotterdam Knowledge Questionnaire for Congenital Heart Disease [31] is used to assess parents’ knowledge about CHD and parents’ illness perception.

##### School functioning

The Dutch version of the Teacher’s Report Form (C-TRF) 1½-5 (100 items) [32] or the TRF/6–18 (120 items) [33] will be completed by the teacher of the child. The TRF assesses problem behavior (at school). Response categories range from 0 to 2, with higher scores indicating more emotional and/or behavioral problems.

##### Executive functioning

The Dutch Behavior Rating Inventory of Executive Functioning (BRIEF) [34, 35] (63 items; 2–5 years) and BRIEF-Preschool version (BRIEF-P) [36] (86 items; 5–18 years) will be used to assess executive functioning skills in daily life. Response categories range from 0 to 2, with higher scores indicating more problems.

##### Enjoyment of leisure-time physical activity

The Groningen Enjoyment Questionnaire (GEQ; 10 items; response categories 1–3, higher score indicates more enjoyment) [37, 38] is adjusted for parents and teachers and assesses enjoyment of physical activity. Children themselves answer two questions to assess how often they engage in physical activity and to assess enjoyment of physical activity.

##### Parental worry

The Penn State Worry Questionnaire (PSWQ; 16 items; response categories 1–5, higher score indicates higher level of worry) [39] assesses the excessiveness and uncontrollability of parental worry.

##### Parenting stress

The Nijmeegse Ouderlijke Stress Index verkort (NOSIK) [40, 41] (25 items; response categories 1–6, higher score indicates higher level of stress) measures stress due to parenting. Parents will also complete the Distress Thermometer (DT-P) [42] (40–42 items), which consists of a problem list and a thermometer on which parents are asked to rate their overall distress.

##### Quality of life of children and siblings

The Child Health Questionnaire Parent Form-50 (CHQ-PF50; 50 items) [43] is used to assess quality of life of the child with CHD and, if possible, of one sibling.

##### Parental quality of life

The Short-form (36) Health Survey (SF-36) [44] (36 items; score per domain 0–100, higher score indicates less disability) assesses eight health status domains: physical functioning, role limitations due to physical problems, bodily pain, general health, social functioning, role limitations due to emotional functioning, mental health, and vitality.

##### Family functioning

The general functioning subscale of the Family Assessment Device (FAD) [45] (12 items; response categories 1–4, higher total score indicates poorer functioning) assesses problem areas of family functioning.

##### Social validity

Through a questionnaire, parents will be asked about their satisfaction regarding CHIP-Family. Furthermore, data on attendance and completion of CHIP-Family will be recorded.

#### Predictors

##### Demographic variables

Demographic variables such as age, gender, and socio-economic status will be assessed through the Rotterdam Quality of Life interview [30].

##### Medical variables

Information about cardiac diagnosis, surgery, and intrusive procedures will be retrieved from medical records.

##### Life events

The ‘life events’ subscale of the Cognitive Emotion Regulation Questionnaire child version (CERQ-k) [44] is adjusted as such that parents can answer the questions about their child.

### Sample size calculation

To conduct a repeated measures ANOVA with two assessment moments, Cohen’s d of 0.6, an alpha of .05 (two-tailed), and a power of .80, a sample size of 90 patients is needed, of which 45 patients in the intervention group.

### Statistical analysis

To test the effectiveness of CHIP-Family on the primary outcome measures (for parents: mental health [SCL-90-R]; for children: behavioral/emotional problems [CBCL]) repeated measures ANOVAs will be conducted, for parental and child outcomes separately. Group (CHIP-Family versus CAU) will be the between-subjects variable and assessment (T1 versus T2) will be the within-subjects variable. Likewise, repeated measures ANOVAs will be conducted for the secondary outcome measures.

Additional regression analyses will be conducted to investigate in what way demographic factors, medical factors, and life events moderate the effect of CHIP-Family on the primary outcome measures.

## Discussion

Several cohort and longitudinal studies have shown that there is a compelling need for a family-based psychosocial intervention for children with CHD and their families [1–3, 18–21]. Since key milestones such as starting kindergarten and primary school present more challenges for children with CHD and their parents than for families with healthy children [20], an intervention tailored to their needs when starting school is needed. The previously examined CHIP-School intervention [2], the only evidence-based psychosocial intervention for this population to date, significantly improved maternal mental health, diminished perceived strain on the family, and resulted in less school absence of the child. However, CHIP-School targeted parents only, aiming for an indirect effect on child psychosocial wellbeing. CHIP-School resulted in a non-significant, though positive, increase in child psychosocial wellbeing.

To improve these outcomes, we will modify and extend CHIP by adding a tailored child module for children with CHD and their siblings, thereby creating the CHIP-Family intervention. The child module consists of evidence-based cognitive behavioral and sports exercises. We will conduct an RCT to examine the effect of the innovated CHIP-Family intervention on parental mental health and psychosocial wellbeing of young children with CHD.

This study has several strengths. Firstly, if CHIP-Family proves to be effective, this would be the first evidence-based psychosocial intervention for young children with CHD and their families, thus meeting the previously described need for an intervention. Secondly, as recommended by the guidelines of the Association for European Pediatric Cardiology working group [22], CHIP-Family provides early intervention. CHIP-Family aims to reduce and prevent psychosocial problems. As mental health problems in childhood may persist into adulthood [22, 46], the prevention of psychosocial problems is important. Thirdly, CHIP-Family is a family-centered intervention. It is widely acknowledged that family functioning and parental factors play an important role in children’s development [2, 14, 15]. As parents of children with CHD are at risk for psychosocial problems [19], a family-centered intervention may reduce their problems [47]. This, in turn, may enhance family functioning. Furthermore, siblings are involved in the workshop and receive attention from the hospital staff, which normalizes the position of the child with CHD.

In conclusion, this intervention aims to fulfill the need for an evidence-based family-centered psychosocial intervention for children with CHD and their families. If CHIP-Family proves to be effective in improving parental mental health and psychosocial wellbeing of children with CHD, it should be structurally implemented in standard care.

## Abbreviations

CAU: Care as usual
CHD: Congenital heart disease
CHIP: Congenital Heart disease Intervention Program
RCT: Randomized controlled trial

## References

[1] Marino BS, Lipkin PH, Newburger JW, Peacock G, Gerdes M, Gaynor JW, Mussatto KA, Uzark K, Goldberg CS, Johnson WH (2012). Neurodevelopmental outcomes in children with congenital heart disease: evaluation and management: a scientific statement from the American Heart Association. Circulation.

[2] McCusker CG, Doherty NN, Molloy B, Rooney N, Mulholland C, Sands A, Craig B, Stewart M, Casey F (2012). A randomized controlled trial of interventions to promote adjustment in children with congenital heart disease entering school and their families. J Pediatr Psychol.

[3] Van Rijen EH, Utens EM, Roos-Hesselink JW, Meijboom FJ, van Domburg RT, Roelandt JR, Bogers AJ, Verhulst FC (2003). Psychosocial functioning of the adult with congenital heart disease: a 20-33 years follow-up. Eur Heart J.

[4] Spijkerboer AW, Utens EM, Bogers AJ, Helbing WA, Verhulst FC (2008). A historical comparison of long-term behavioral and emotional outcomes in children and adolescents after invasive treatment for congenital heart disease. J Pediatr Surg.

[5] Utens EM, Verhulst FC, Meijboom FJ, Duivenvoorden HJ, Erdman RA, Bos E, Roelandt JT, Hess J (1993). Behavioural and emotional problems in children and adolescents with congenital heart disease. Psychol Med.

[6] Karsdorp PA, Everaerd W, Kindt M, Mulder BJ (2007). Psychological and cognitive functioning in children and adolescents with congenital heart disease: a meta-analysis. J Pediatr Psychol.

[7] Menahem S, Poulakis Z, Prior M (2008). Children subjected to cardiac surgery for congenital heart disease. Part 1 - emotional and psychological outcomes. Interact Cardiovasc Thorac Surg.

[8] Bellinger DC, Newburger JW (2010). Neuropsychological, psychosocial, and quality-of-life outcomes in children and adolescents with congenital heart disease. Prog Pediatr Cardiol.

[9] Wernovsky G (2006). Current insights regarding neurological and developmental abnormalities in children and young adults with complex congenital cardiac disease. Cardiol Young.

[10] Opic P, Roos-Hesselink JW, Cuypers JA, Witsenburg M, van den Bosch A, van Domburg RT, Bogers AJ, Utens EM (2015). Psychosocial functioning of adults with congenital heart disease: outcomes of a 30-43 year longitudinal follow-up. Clin Res Cardiol.

[11] Zomer AC, Vaartjes I, Uiterwaal CS, van der Velde ET, Sieswerda GJ, Wajon EM, Plomp K, van Bergen PF, Verheugt CL, Krivka E (2012). Social burden and lifestyle in adults with congenital heart disease. Am J Cardiol.

[12] Dulfer K, Helbing WA, Duppen N, Utens EMWJ (2014). Associations between exercise capacity, physical activity, and psychosocial functioning in children with congenital heart disease: a systematic review. Eur J Prev Cardiol.

[13] Dulfer K, Duppen N, Kuipers IM, Schokking M, van Domburg RT, Verhulst FC, Helbing WA, Utens EM (2014). Aerobic exercise influences quality of life of children and youngsters with congenital heart disease: a randomized controlled trial. J Adolesc Health.

[14] McCusker CG, Doherty NN, Molloy B, Rooney N, Mulholland C, Sands A, Craig B, Stewart M, Casey F (2010). A controlled trial of early interventions to promote maternal adjustment and development in infants born with severe congenital heart disease. Child Care Health Dev.

[15] Jackson AC, Liang RPT, Frydenberg E, Higgins RO, Murphy BM (2016). Parent education programmes for special health care needs children: a systematic review. J Clin Nurs.

[16] Landolt MA, Ystrom E, Stene-Larsen K, Holmstrom H, Vollrath ME (2014). Exploring causal pathways of child behavior and maternal mental health in families with a child with congenital heart disease: a longitudinal study. Psychol Med.

[17] Casey FA, Stewart M, McCusker CG, Morrison ML, Molloy B, Doherty N, Craig BG, Sands AJ, Rooney N, Mulholland HC (2010). Examination of the physical and psychosocial determinants of health behaviour in 4-5-year-old children with congenital cardiac disease. Cardiol Young.

[18] Lawoko S, Soares JJ (2003). Quality of life among parents of children with congenital heart disease, parents of children with other diseases and parents of healthy children. Qual Life Res.

[19] Lawoko S, Soares JJ (2006). Psychosocial morbidity among parents of children with congenital heart disease: a prospective longitudinal study. Heart Lung.

[20] Tong EM, Kools S (2004). Health care transitions for adolescents with congenital heart disease: patient and family perspectives. Nurs Clin North Am.

[21] Lesch W, Specht K, Lux A, Frey M, Utens E, Bauer U. Disease-specific knowledge and information preferences of young patients with congenital heart disease. Cardiol Young. 2014;24(2):1–10. 10.1017/S1047951113000413 23628281

[22] Utens E, Callus E, Levert E, De Groote K, Casey F. Multidisciplinary family-centred psychosocial care for patients with CHD: consensus recommendations from the AEPC psychosocial working group. Cardiol Young. 2017;28(2):1–7. 10.1017/S1047951117001378 28889827

[23] Levert EM, Helbing WA, Dulfer K, van Domburg RT, Utens EM. Psychosocial needs of children undergoing an invasive procedure for a CHD and their parents. Cardiol Young. 2016;27(2):1–12. 10.1017/S1047951116000391 27055357

[24] Spijkerboer AW, Utens EMWJ, Bogers AJJC, Verhulst FC, Helbing WA (2008). Long-term intellectual functioning and school-related behavioural outcomes in children and adolescents after invasive treatment for congenital heart disease. Br J Dev Psychol.

[25] Pahl KM, Barrett PM (2010). Preventing anxiety and promoting social and emotional strength in preschool children: a universal evaluation of the fun FRIENDS program. Adv School Ment Health Promot.

[26] Schulz KF, Altman DG, Moher D (2010). CONSORT 2010 statement: updated guidelines for reporting parallel group randomised trials. J Pharmacol Pharmacother.

[27] Achenbach TM, Rescorla LA (2001). Manual for the ASEBA school-age forms & profiles.

[28] Achenbach TM, Becker A, Dopfner M, Heiervang E, Roessner V, Steinhausen HC, Rothenberger A (2008). Multicultural assessment of child and adolescent psychopathology with ASEBA and SDQ instruments: research findings, applications, and future directions. J Child Psychol Psychiatry.

[29] Arrindell WA, Ettema JHM (2003). SCL-90. Symptom checklist. Handleiding bij een multidimensionele psychopathologie-indicator.

[30] Utens EMWJ, Dulfer K (2010). Rotterdams Kwaliteit van Leven Interview.

[31] Utens EMWJ, Dulfer K (2010). Rotterdam knowledge questionnaire.

[32] Achenbach TM, Rescorla LA (2000). Manual for ASEBA Preschool Forms & Profiles.

[33] Verhulst FC, van der Ende J (2013). Handleiding ASEBA Vragenlijsten voor leeftijden 6 tot en met 18 jaar.

[34] Smidts DP, Huizinga M (2009). BRIEF Executieve Functies Gedragsvragenlijst: Handleiding.

[35] Gioia GA, Isquith PK, Guy SC, Kenworthy L (2000). Behavior rating inventory of executive function.

[36] Van der Heijden KB, Suurland J, De Sonneville LMJ, Swaab HJT (2013). BRIEF-P Vragenlijst executieve functies voor 2- tot 5-jarigen.

[37] Stevens M, Moget P, de Greef MH, Lemmink KA, Rispens P (2000). The Groningen enjoyment questionnaire: a measure of enjoyment in leisure-time physical activity. Percept Mot Skills.

[38] Dulfer K, Duppen N, Blom NA, van Dijk AP, Helbing WA, Verhulst FC, Utens EM (2014). Effect of exercise training on sports enjoyment and leisure-time spending in adolescents with complex congenital heart disease: the moderating effect of health behavior and disease knowledge. Congenit Heart Dis.

[39] Van der Heiden C, Muris P, Bos AE, van der Molen HT (2010). Factor structure of the Dutch version of the Penn State worry questionnaire. J Behav Ther Exp Psychiatry.

[40] Brock AJLL, Vermulst AA, Gerris JRM, Abidin RR (1992). NOSI, Nijmeegse Ouderlijke Stress Index, handleiding.

[41] Abidin RR (1983). Parenting stress index.

[42] Haverman L, van Oers HA, Limperg PF, Houtzager BA, Huisman J, Darlington AS, Maurice-Stam H, Grootenhuis MA (2013). Development and validation of the distress thermometer for parents of a chronically ill child. J Pediatr.

[43] Langraf JM, Abetz L, Ware JE (1999). The CHQ user’s manual.

[44] Garnefski N, Rieffe C, Jellesma F, Terwogt MM, Kraaij V (2007). Cognitive emotion regulation strategies and emotional problems in 9 - 11-year-old children: the development of an instrument. Eur Child Adolesc Psychiatry.

[45] Epstein NB, Baldwin LM, Bishop DS (1983). The McMaster family assessment device. J Marital Fam Ther.

[46] Hofstra MB, van der Ende J, Verhulst FC (2002). Child and adolescent problems predict DSM-IV disorders in adulthood: a 14-year follow-up of a Dutch epidemiological sample. J Am Acad Child Adolesc Psychiatry.

[47] Law EF, Fisher E, Fales J, Noel M, Eccleston C (2014). Systematic review and meta-analysis of parent and family-based interventions for children and adolescents with chronic medical conditions. J Pediatr Psychol.

[48] D’Zurilla TJ, Nezu AM, Dobson KS (2010). Problem-solving therapy. Handbook of cognitive-behavioral therapies.

[49] Meyer TJ, Miller ML, Metzger RL, Borkovec TD (1990). Development and validation of the Penn State worry questionnaire. Behav Res Ther.

[50] Tuinman MA, Gazendam-Donofrio SM, Hoekstra-Weebers JE (2008). Screening and referral for psychosocial distress in oncologic practice: use of the distress thermometer. Cancer.

[51] Hullmann SE, Ryan JL, Ramsey RR, Chaney JM, Mullins LL (2011). Measures of general pediatric quality of life: child health questionnaire (CHQ), DISABKIDS chronic generic measure (DCGM), KINDL-R, pediatric quality of life inventory (PedsQL) 4.0 generic Core scales, and quality of my life questionnaire (QoML). Arthritis Care Res.

[52] Aaronson NK, Muller M, Cohen PD, Essink-Bot ML, Fekkes M, Sanderman R, Sprangers MA, te Velde A, Verrips E (1998). Translation, validation, and norming of the Dutch language version of the SF-36 health survey in community and chronic disease populations. J Clin Epidemiol.

[53] Epstein L (1983). Family and community medicine: complementary components of primary health care. Isr J Med Sci.

